# Serum levels of MMP3 could be used as a potential biomarker for sports-induced knee osteoarthritis

**DOI:** 10.5937/jomb0-52021

**Published:** 2026-02-27

**Authors:** Shanling Gu, Yan Xu, Xiaowei Yang

**Affiliations:** 1 The First Hospital of Jilin University, Jilin University, Changchun, China

**Keywords:** serum, MMP3, biomarker, sports, knee osteoarthritis, serum, MMP3, biomarker, sport, osteoartritis kolena

## Abstract

**Background:**

Evaluating the potential biomarkers for diagnosing knee osteoarthritis caused by sports activities.

**Methods:**

This was a body-split case-control study. We identified 25 eligible participants for our research with no other knee procedures besides meniscus removal. For the determination of serum Glycosaminoglycan (GAG) and matrix metalloproteinase 3 (MMP-3) levels, blood was taken from 25 eligible individuals using 3 simple 6 mL EDTA vacutainer tubes. Patients also had knee aspirations, both surgical and non-surgical. Out of the 25 attempts, a successful knee synovial fluid aspirate was obtained. Additionally, all 25 attempts to get a contralateral control knee aspirate were successful. The raw values of the cohorts' demographics, radiological, clinical, PROMs, and biomarkers of MMP3 and GAG.

**Results:**

The duration of pain was 4.52 ± 0.85 and 5.85 ± 1.22 in non-operated and operated individuals, respectively. The VSA Pain score was 48.74 ± 2.87 and 53.55 ± 3.39, and the Lequesne algofunctional index was 11.02 ± 1.29 and 13.36 ± 1.57 in non-operated and operated participants, respectively. A comparative examination of MMP-3 levels in the synovial fluid showed that the levels of MMP3 remain notably elevated in the affected knees (p= 0.01). Additionally, synovial GAG levels in the affected knee joint were dramatically reduced (p= 0.03). An inverse connection was seen between the levels of MMP-3 and GAGs in the synovial fluid when analysing all 25 samples from both non-affected and affected knees. The correlation coefficient (r) was -0.88, indicating a strong negative relationship. The p-value was 0.04, suggesting statistical significance.

**Conclusions:**

Our results suggest that MMP3 serum levels may be a simple blood test away from becoming a diagnostic tool for knee osteoarthritis and a possible predictor of the disease. To better assess athletes' joint health, it may be helpful to combine biomarkers with other diagnostic tools and have a comprehensive understanding of their athletic background.

## Abstract (sr)

**Uvod:**

Evaluacija potencijalnih biomarkera za dijagnostikovanje osteoartritisa kolena izazvanog sportskim aktivnostima.

**Metode:**

Ovo je bila studija tipa slučaj-kontrola sa pregledom situacije na obe strane tela. Identifikovali smo 25 učesnika koji su ispunjavali uslove za naše istraživanje bez drugih procedura na kolenu osim uklanjanja meniskusa. Za određivanje nivoa glikozaminoglikana (GAG) i matriks metaloproteinaze 3 (MMP-3) u serumu uzeti su uzorci krvi od 25 osoba koje su ispunjavale uslove, koristeći tri jednostavne vakutajner epruvete od 6 ml sa EDTA. Pacijenti su takođe imali aspiracije kolena, hirurške i nehirurške. Uspešan uzorak sinovijalne tečnosti kolena je dobijen u 25 slučajeva. Pored toga, bilo je uspešno i svih 25 pokušaja dobijanja uzorka kontrolnog kolena na suprotnoj strani tela. Prikazani su sirovi podaci o demografiji, radiološkim i kliničkim parametrima, PROM-ovima i biomarkerima MMP-3 i GAG za obe grupe.

**Rezultati:**

Trajanje bola je bilo 4,52 ± 0,85 i 5,85 ± 1,22 kod neoperisanih i operisanih osoba, respektivno. VSA skor za bol je bio 48,74 ± 2,87 i 53,55 ± 3,39, a "Lequesne" algofunkcionalni indeks je bio 11,02 ± 1,29 i 13,36 ± 1,57 kod neoperisanih i operisanih učesnika, respektivno. Uporedno ispitivanje nivoa MMP-3 u sinovijalnoj tečnosti pokazalo je da nivoi MMP3 ostaju značajno povišeni u zahvaćenim kolenima (p= 0,01). Pored toga, nivoi sinovijalnog GAG-a u zahvaćenom zglobu kolena su dramatično smanjeni (p= 0,03). Uočena je inverzna veza između nivoa MMP-3 i GAG-a u sinovijalnoj tečnosti kada je analizirano svih 25 uzoraka i sa nezahvaćenih i sa zahvaćenih kolena. Koeficijent korelacije (r) je bio -0,88, što ukazuje na jaku negativnu vezu. P-vrednost je bila 0,04, što ukazuje na statistički značaj.

**Zaključak:**

Naši rezultati ukazuju da su nivoi seruma MMP3 jednostavan test krvi koji može postati dijagnostički alat za osteoartritis kolena ili mogući prediktor bolesti. Da bi se bolje procenilo zdravlje zglobova sportista, treba kombinovati biomarkere sa drugim dijagnostičkim alatima, a potrebno je i da se sveobuhvatno sagleda istorijat bavljenja sportskim aktivnostima.

## Introduction

One of the leading causes of pain and disability in the elderly is osteoarthritis (OA), the most common form of joint disease. Although OA is usually associated with human ageing, it may develop due to several other major risk factors. Some of these factors include being overweight, being a woman, having a history of repetitive stress injuries or trauma to the joints, having weak muscles, a family history of metabolic disorders, a history of rheumatoid arthritis (RA), a history of infections in the joints, crystal deposition (gout), and other disorders involving blood clotting and bone turnover [1][2].

Sports-induced knee osteoarthritis is the occurrence or progression of osteoarthritis in the knee joint due to sports-related activities or accidents. Prolonged strain and excessive use of the knee joint may expedite the deterioration of the cartilage, resulting in osteoarthritis. sSports that require continuous running, leaping, and rotating, such as basketball, soccer, and tennis, may lead to such a condition. Acute injuries to the knee, such as ligament tears or fractures, might elevate the likelihood of developing osteoarthritis in the future. Athletes who suffer from such injuries are more susceptible to joint deterioration [3][4].

Implementing injury prevention programmes prioritising the development of muscular strength, enhancement of flexibility, and total joint stability. Treatment may include a blend of adjustments to one's lifestyle, physical therapy, drugs, and, in some instances, surgical procedures [5].

Measurable indicators of biological processes exist, including anatomical, physiological, biochemical, or molecular aspects [6]. However, radiography insufficiently assesses OA due to limited, accurate clinical assessment ability. Also, often, no association exists between radiographic findings and clinical symptoms, particularly early-stage OA. This shows how radiographic imaging hindered OA research efforts [7]. Due to accurate early-stage display of all key OA-affected joint tissues, magnetic resonance imaging (MRI) is increasingly gaining acknowledgement. MRI's significance in OA diagnosis and prognosis is seen as being of progressive emphasis. Still, early-stage identification of joint structural changes via MRI does not always mean clinically diagnosed OA, especially symptomless [8][9][10]. At the same time, there is no approved biomarker for OA [11][12][13][14][15][16]. Recent studies have shown the role of matrix metalloproteinases (MMPs) in the pathogenesis of osteoporosis. MMPs are a family of proteases involved in the degradation of the extracellular matrix and play a crucial role in bone remodelling [17][18][19]. Specifically, MMP-3 has been identified as a key player in bone resorption and linked to osteoporosis [17][18][19]. Studies have shown that MMP-3 regulates bone metabolism and can influence bone mass and remodelling [17][18][19]. As current diagnostic methods for sports-induced knee OA are limited by their inability to detect early-stage disease and monitor treatment efficacy accurately, we aimed to investigate the potential of serum MMP3 levels as a novel biomarker for knee OA diagnosis and prognosis. Notably, our study addresses the existing gap in the literature by exploring the relationship between serum MMP3 levels and knee OA, which has not been extensively examined in previous studies.

## Material and methods

### Study design

This split-body case-control study investigated the relationship between matrix metalloproteinase-3 (MMP-3) levels and knee osteoarthritis. Before enrollment in the study, written informed consent was obtained from all participants. Participants were provided with a detailed explanation of the study procedures, risks, and benefits and assured of their right to withdraw from the study at any time. The informed consent form addressed the risks and benefits of knee aspiration from the affected and contralateral knee.

### Participant selection

Eighty patients who had undergone radiographic evaluation of both knees were initially recruited for this study. The sample size was calculated using a power analysis to detect a significant difference in MMP-3 levels between cases and controls. Assuming a moderate effect size (Cohen's d=0.5) and a significance level of 0.05, a sample size of 48 patients per group was sufficient to detect a significant difference in MMP-3 levels between cases and controls. Simple, consecutive sampling was used to select participants for the study. Patients who met the inclusion criteria and provided informed consent were enrolled until the desired sample size was reached.

### Sample collection

Blood samples were collected from the 25 eligible patients using 3 simple 6 mL EDTA vacutainer tubes. Knee aspirations were also performed on these patients, with 50 successful synovial fluid aspirates obtained from the affected knee and 50 successful contralateral control knee aspirates.

### Sample preparation

Clinical and radiographic evaluations were performed on all participants to assess the severity of knee osteoarthritis. Clinical parameters, including pain duration (years), pain severity (visual analogue scale, VAS, in mm), and disease severity (Lequesne's algofunctional index), were recorded. Then, undiluted aspirate samples were placed into plain tubes and transported to the biochemistry laboratory on-site. The samples were centrifuged and stored at -70°C to prevent damage from repeated freezing and thawing.

### MMP-3 quantification

MMP-3 levels were quantified using enzyme-linked immunosorbent assay (ELISA). The ELISA kit was used according to the manufacturer's instructions. Briefly, the kit reagents and samples were equilibrated to room temperature, and the sample diluent was prepared if not provided in the kit. The MMP-3 standard was prepared, and serial dilutions were performed to generate a standard curve. The plate was coated with MMP-3 antibody, washed, and blocked with a blocking buffer. Samples and standards were added to the wells, incubated, and washed. The antibody was added, followed by substrate solution, and the reaction stopped. The plate was read using a microplate reader, and MMP-3 concentrations were determined by comparing sample absorbance to the standard curve [20].

### GAG quantification

Glycosaminoglycan (GAG) levels were quantified using a modified Bjornsson Alcian blue precipitation technique. Briefly, samples and chondroitin sulphate standards were heated to 4°C and left to precipitate for two hours in a solution containing Alcian blue, guanidinium hydrochloride, Triton X-100, and H_2_SO_4_. The particles were resuspended in guanidinium hydrochloride and 1-propanol, and the absorbance was measured at 600 nm [21].

### Radiographic examination

Radiographs of the knees were taken with the subject standing, and weight was applied to each knee at an angle of 15 degrees. Two investigators (IP AA) independently evaluated the radiographs for knee tibiofemoral joint (TFJ) OA using the Kellgren-Lawrence and Ahlback scoring systems [22][23]. Scoring was performed without magnifying equipment from a distance of 60 cm. Radiographic evaluation was performed using MRI, and the severity of knee osteoarthritis was graded according to the Kellgren-Lawrence (KL) classification system, which ranges from KL0 (normal) to KL4 (severe osteoarthritis). A blinded radiologist assessed the KL grades, and the results were categorised into KL1, KL2, and KL3. The data were compared between the non-operated and operated groups to identify significant clinical and radiographic parameter differences.

### Statistical examination

Correlations were examined using Pearson's rank correlation coefficient as part of the investigation. We considered p-values less than 0.05 to be statistically significant. Meniscectomy patients with elevated serum MMP-3 levels likely also have elevated synovial fluid MMP-3 levels. There was a clear correlation between the blood MMP-3 levels and the treated joints' synovial fluid MMP-3 levels. However, there was a noticeable difference in the pattern of MMP-3 concentrations between the operated and non-operated joints. Consequently, serum MMP-3 levels may be a suitable alternative for synovial MMP-3 levels in these knees that have undergone surgery.

## Results

After excluding 10 patients who had undergone complete knee replacement, 5 who had died, 5 who were no longer being monitored, and 5 who were unable to attend clinical assessment, 55 patients were eligible for clinical evaluation. Five patients who had undergone knee procedures on the opposite side were excluded, leaving 25 patients who had not undergone any other knee procedures apart from meniscus removal.

Table 1 indicates that the majority of the participants, namely 82%, were male, while just 18% were female. The majority of participants, 66%, belonged to the age range of 40-50 years, followed by 20% in the 50-60 age group, 12% beyond 60 years of age, and 2% below 30 years of age. The average age of the participants was 40.58±5.85. The measurements for body height, BMI, and sports activity per week were recorded as 180.58±2.85 cm, 23.58±2.55 kg/m^2^, and 4.51±1.33 hours, respectively.

**Table 1:** Basic profile of the participants.

	Number=25	Percentage/SD
Gender		
Male	41	82
Female	9	18
** Age **		
30-40	1	2
40-50	33	66
50-60	10	20
Above 60	6	12
Mean Age	40.58	5.85
Body height (cm)	180.58	2.85
BMI (kg/m^2^)	23.58	2.55
Sports activity per week (hours)	4.51	1.33

**Figure 1:**
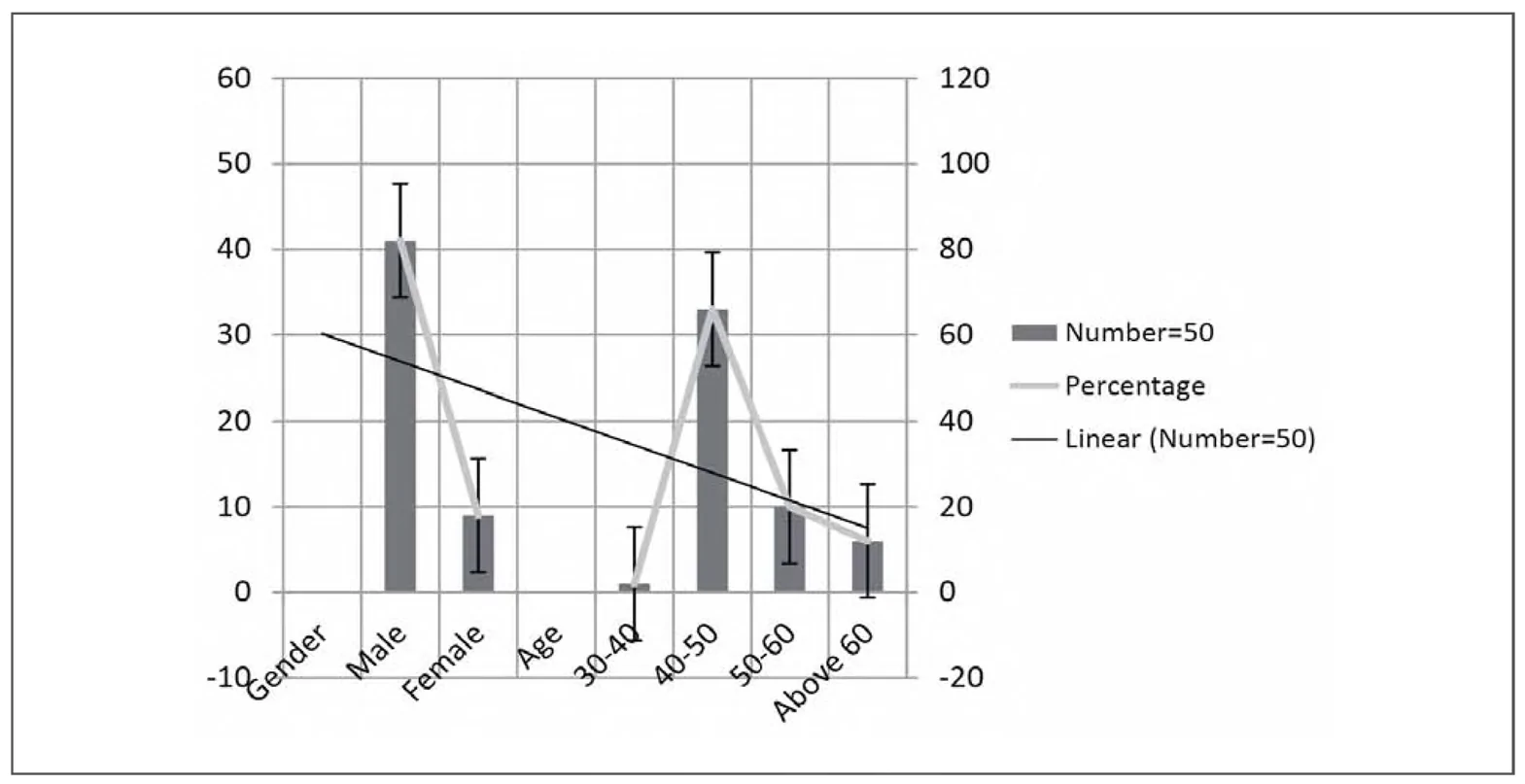
Basic profile of the participants.

**Figure 2:**
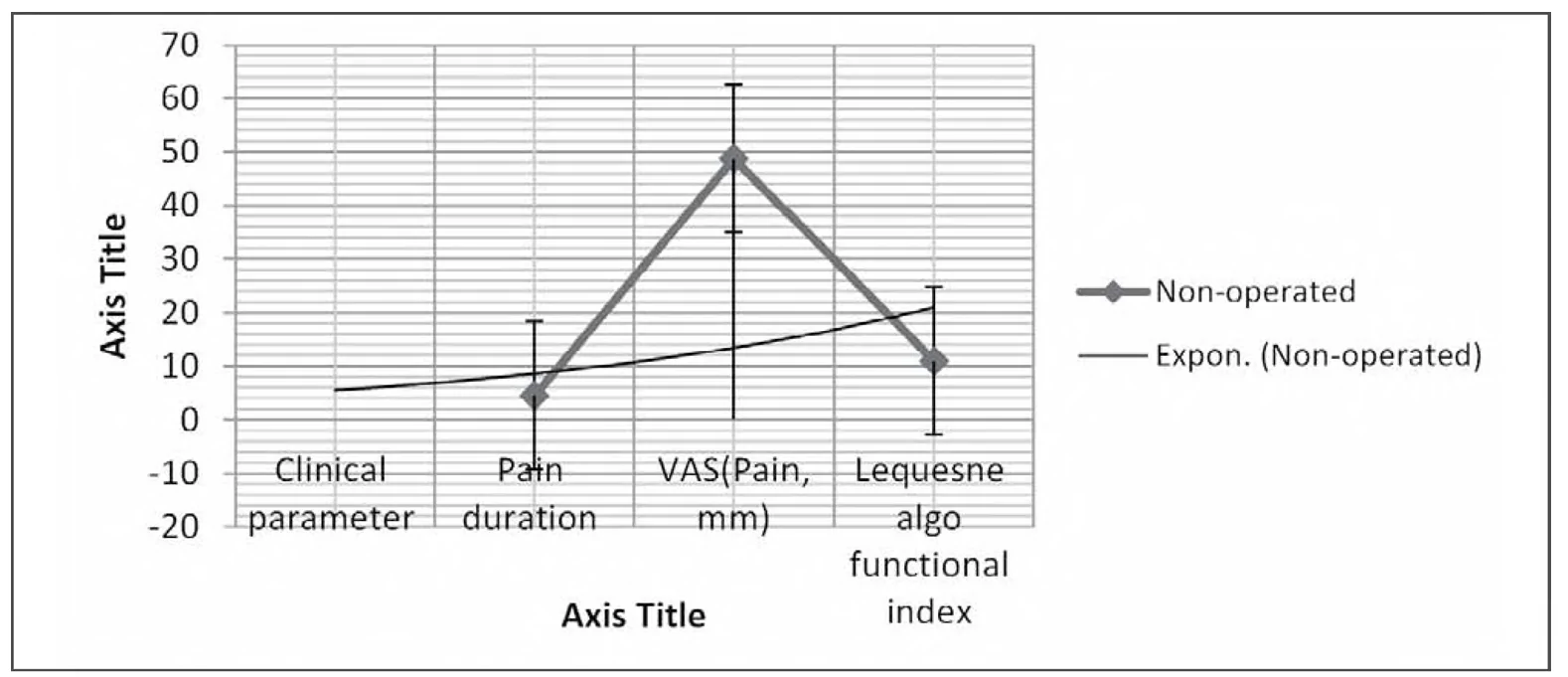
Clinical parameter of the participants.

The clinical and MRI parameters of the subjects are shown in Table 2. The duration of pain was 4.52±0.85 and 5.85±1.22 in non-operated and operated individuals, respectively. The VSA Pain score was 48.74±2.87 and 53.55±3.39, and the Lequesne algofunctional index was 11.02 ±1.29 and 13.36±1.57 in non-operated and operated participants, respectively. The MRI parameter values for non-operated and operated subjects were as follows: KL1 was 10 and 11, KL2 was 10 and 11, and KL3 was 4 and 4 (Table 2). Figure 1 Figure 2

**Table 2:** Clinical and MRI parameters of the participants. KL, Kellgren-Lawrence classification

	Non-affected	affected	p-value
Clinical parameter			
Pain duration	4.52±0.85	5.85±1.22	0.36
VAS(Pain, mm)	48.74±2.87	53.55±3.39	0.47
Lequesne algofunctional index (disease severity)	11.02±1.29	13.36±1.57	0.15
MRI parameter			
KL1	10	11	0.11
KL2	10	11	0.34
KL3	4	4	0.14

**Figure 3:**
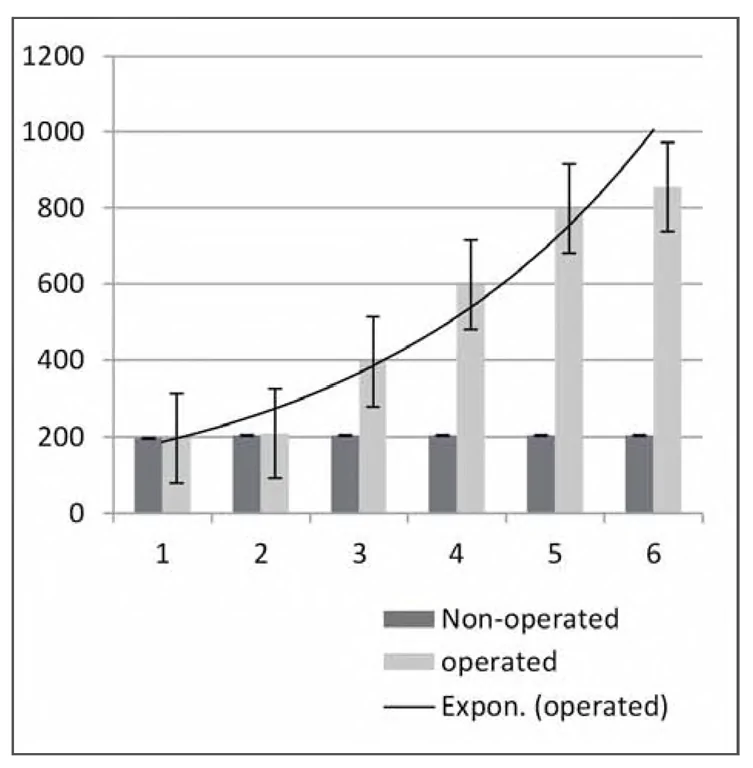
MMP-3 Protein level in synovial fluid.

**Figure 4:**
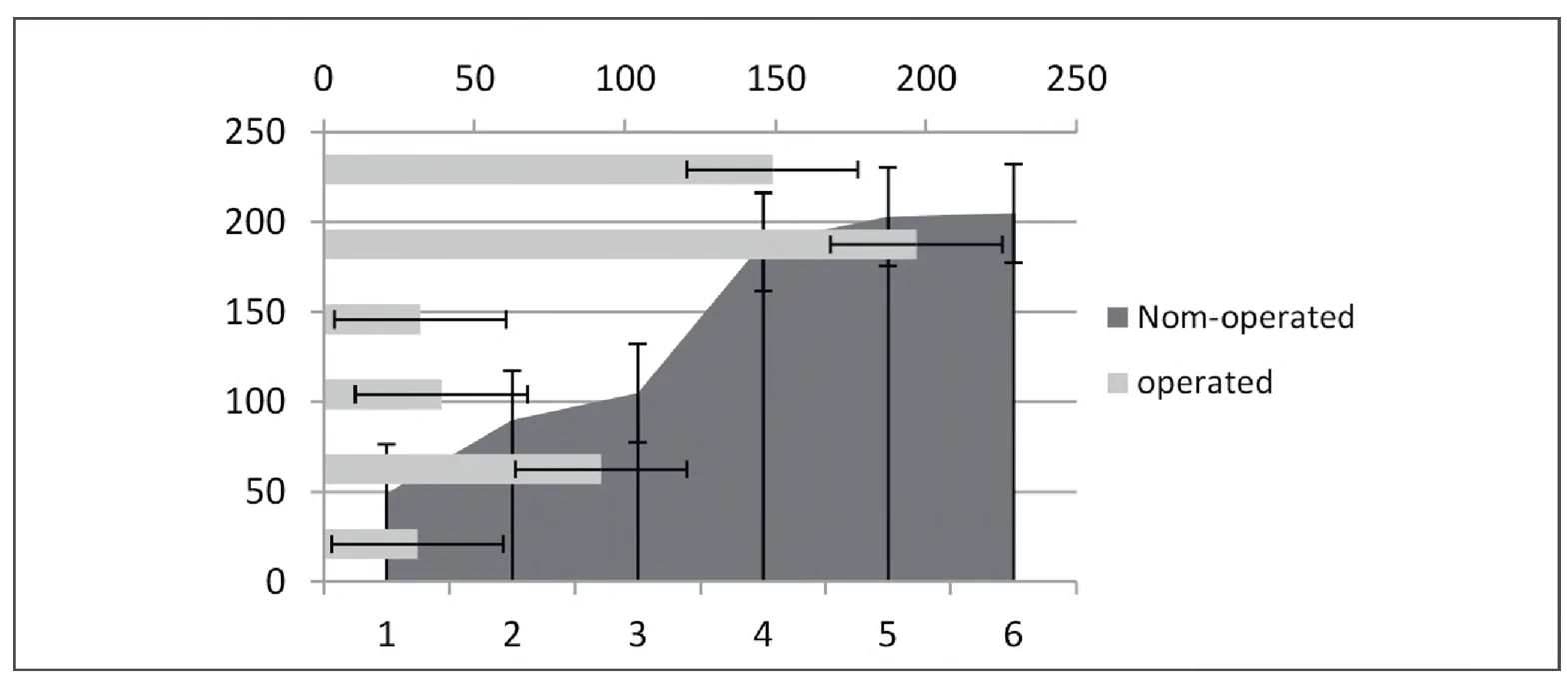
GAG protein levels wirhin the synovial fluid.

We compared the operated and non-operated knees. We conducted a comparative analysis of MMP-3 and GAG levels in the knees of 25 patients with successful bilateral knee synovial fluid aspirations, comparing the knees. A comparative examination of MMP-3 levels in the synovial fluid showed that, despite the considerable time elapsed after the operation, the levels of MMP3 remain notably elevated in the operated knee (p=0.01) (Table 3 and Figure 3). Additionally, the levels of synovial GAGs in the operated joint were dramatically reduced (p=0.03) (Table 4 and Figure 4).

**Table 3:** MMP-3 Protein level in synovial fluid.

MMP-3 Protein level in synovial fluid	Non-affected	affected	r-value	p-value
1	197	197		
2	205	210	0.05	0.01
3	205	397		
4	205	598		
5	205	798		
6	205	855		

**Table 4:** GAG protein levels within the synovial fluid.

Synovial GAGs levels	Non-affected	affected	r-value	p-value
1	49	31		
2	90	92	0.07	0.03
3	105	39		
4	189	32		
5	203	197		
6	205	149		

When analysing all 50 samples from both operated and non-operated knees, an inverse connection was seen between the levels of MMP-3 and GAGs in the synovial fluid. The correlation coefficient (r) was -0.88, indicating a strong negative relationship. The p-value was 0.04, suggesting statistical significance. These findings are summarised in Table 5.

**Table 5:** Correlation of absolute GAG levels and absolute MMP-3 levels in the synovial fluid of all knees.

	MMP-3	GAG	R-value	P-value
1	585	20		
2	500	50	0.08	0.04
3	400	100		
4	350	150		
5	250	200		
6	200	250		

Table 6 displays the average values and variability, as measured by standard deviations, of GAG and MMP-3 concentrations.

**Table 6:** MMP3 content and GAG content in non-operated and operated participants.

Outcome	Non-affected		affected		r-value	p-value
	Mean	Sd	Mean	Sd		
MMP3 content	281.55	13.36	451.58	29.96		0.02
GAG content	161.58	25.85	121.47	7.29		0.03

**Figure 5:**
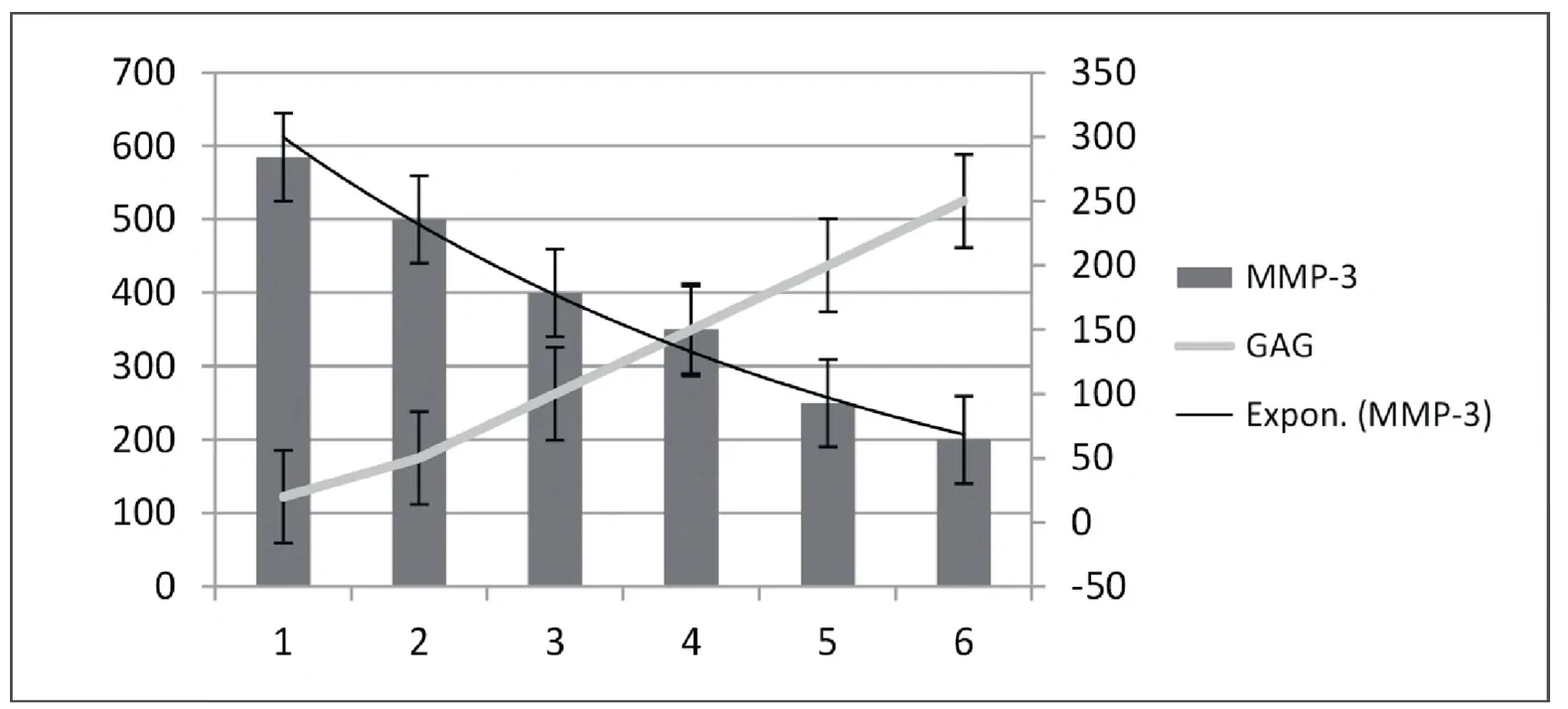
Correlation of absolute GAG levels and absolute MMP-3 levels in the synovila fluid all knees.

**Figure 6:**
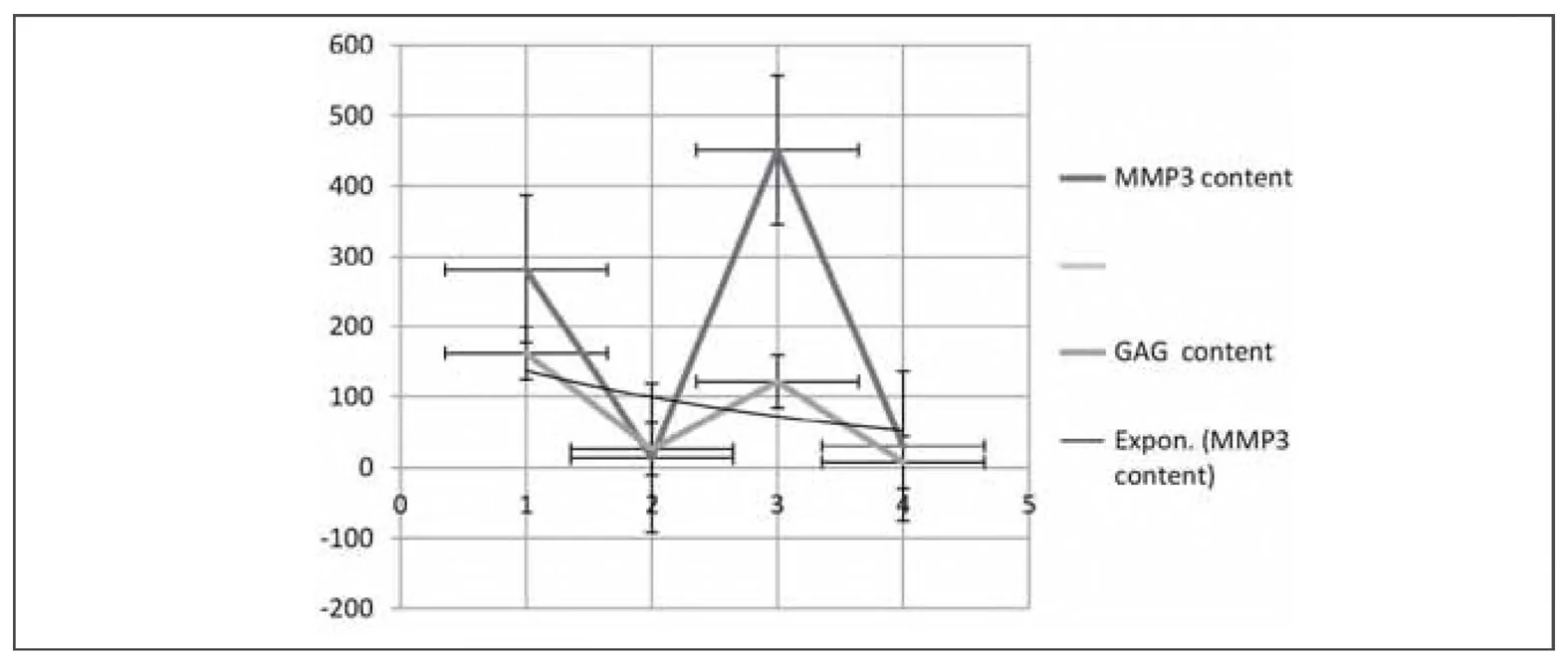
MMP3 content and GAG content in non operated and operated participants.

Radiographs show that synovial MMP-3 levels are up while GAG levels are lowered. According to the research, there was a robust positive relationship between the MMP-3 levels in the knee synovial fluid and the Ahlb ck and Kellgren-Laurence (KL) radiographic assessment systems (p=0.004, r=0.96; p 0.02, r=-0.77). Alternatively, the Ahlb ck and Kellgren-Laurence (KL) radiographic assessment systems were found to have a robust negative association with the levels of GAG in the knee synovial fluid (p=0.03, r=0.63; p=0.02, r=-0.74). Quality of life was also predicted by patient age and MMP-3 levels using a multiple regression model. Figure 5 Figure 6

The quality of life was determined using stepwise regression analysis and a prediction model that used clinical and biochemical data. Age at surgery, GAG levels 20 years after surgery, variation in GAG levels from baseline, and amount of MMP-3 levels 20 years after surgery were all factors incorporated in the final model. In Table 7 and Figure 7, you can see the coefficients. With a p-value of 0.005 and an R2-value of 0.98, the model demonstrated remarkable accuracy in predicting QOL. The results show that these traits add to a higher quality of life.

**Table 7:** Coefficients of multiple regression analysis for patient quality of life.

	Estimate	Std. error	t-value	Pr(>|t)
(Intercept)	-1.58	2.52	-31.25	0.03
Age At Sur	1.29	3.02	41.29	0.02
Gagind40	4.15	4.01	111.08	0.01
dGAG40	-2.33	3.07	-82.54	0.01
MMP3ind40	-1.74	2.58	-59.96	0.01

## Discussion

We compared the operated and non-operated knees. We conducted a comparative analysis of MMP-3 and GAG levels in the knees of 25 patients with successful bilateral knee synovial fluid aspirations, comparing the knees. Our study found that serum levels of MMP-3 were significantly higher in OA patients than controls. Based on the search results, it appears that there is a significant association between matrix metalloproteinase-3 (MMP-3) and osteoarthritis (OA). Several studies have investigated the role of MMP-3 in OA, and the results suggest that MMP-3 is upregulated in the synovium and chondrocytes of OA patients, contributing to the progression of cartilage lesions [23][24]. Additionally, genetic polymorphisms of MMP-3 have been associated with an increased risk of knee OA, particularly in Asian populations [25][20].

Singh et al. [26] and Jarecki et al. [27] studies found that MMP3 levels were elevated in osteoarthritis patients compared to controls, which was entirely confirmed by our research. Singh et al. [26] and Jarecki et al. [27] found that MMP3 levels increased with disease severity, while our study found that MMP3 levels were elevated in affected knees. The correlations between MMP3 levels and age, BMI, KL grade, and synovial pro-MMP13 levels [26][27] suggest that MMP3 may be a valuable biomarker for monitoring disease progression and response to treatment.

Our study, a split-body case-control study, and the study by Chen et al. [28] share similarities in investigating the role of MMP-3 in OA, using knee synovial tissue/fluid as the sample source, and aiming to understand the relationship between MMP-3 levels and the severity of OA. However, differences exist in study design, with our study using a split-body design and Chen et al.'s study using a case-control design with three subgroups of OA patients. Methodological differences exist, with our study using enzyme-linked immunosorbent assay (ELISA) to quantify MMP-3 levels and Chen et al.'s [28] study using immunohistochemical assay. Our study provides a more comprehensive understanding of the relationship between MMP-3 levels and various clinical, radiographic, and biochemical parameters, including quality of life. In contrast, Chen et al.'s [28] study focuses on the expression of MMP-3 protein in knee synovial tissue and its correlation with OA severity.

A comparative analysis of our study and Sulastri et al.'s [29] investigation reveals distinct approaches to elucidating the role of MMP-3 in OA [29]. While our research employs a split-body case-control design to explore the interplay between MMP-3 levels and various clinical, radiographic, and biochemical parameters, Sulastri et al.'s study adopts a cross-sectional approach to identify risk factors associated with elevated MMP-3 gene expression in knee OA patients. Notably, Sulastri et al.'s [29] study highlights the significance of MMP-3 gene polymorphism rs679620, age, and IL-1 and TNF-α levels as predictors of increased MMP-3 gene expression. In contrast, our study underscores the importance of MMP-3 levels in modulating GAG levels and influencing patient outcomes.

## Conclusions

The study's findings suggest that MMP3 plays a crucial role in the pathophysiology of osteoarthritis. Identifying MMP3 as a potential biomarker in serum levels offers a promising avenue for developing a non-invasive diagnostic tool. By identifying MMP3 as a biomarker, healthcare professionals may be able to detect knee osteoarthritis at an earlier stage, allowing for timely interventions that can slow or prevent disease progression. While this study focused on sports-induced osteoarthritis, the findings may have broader implications for diagnosing and managing osteoarthritis.

## Dodatak

### Conflict of interest statement

All the authors declare that they have no conflict of interest in this work.
